# Wake-Independent Velocity Estimation and Motion Compensation for SAR Moving Target Based on Time–Frequency Analysis

**DOI:** 10.3390/s26030832

**Published:** 2026-01-27

**Authors:** Chun Wen, Yunhua Wang, Yanmin Zhang, Honglei Zheng, Daozhong Sun, Qian Li, Fei Chen

**Affiliations:** 1 Faculty of Information Science and Engineering, Ocean University of China, Qingdao 266100, China; 2 Hangzhou Institute of Technology, Xidian University, Hangzhou 311231, China

**Keywords:** SAR imaging, moving ship target, time–frequency analysis, velocity estimation, motion compensation

## Abstract

**Highlights:**

**What are the main findings?**

- A wake-independent velocity estimation and motion compensation framework based on time–frequency analysis is proposed, where the beam center crossing time is determined by detecting abrupt intensity transitions.
- The method is verified using both simulation results and Sentinel-1 data.

**What are the implications of the main findings?**

- It provides a more general motion compensation technique for sea surface ship targets, especially for targets without significant wake.
- The successful application on Sentinel-1 data validates its feasibility for maritime mobile target monitoring.

**Abstract:**

Imaging moving targets in synthetic aperture radar (SAR) remains a significant challenge due to the defocusing and azimuthal displacement caused by target motion. To address this, this paper proposes a velocity estimation and motion compensation technique to mitigate the impact of moving targets on SAR imaging quality. The core innovation of this study lies in a wake-independent method for determining the radar beam center crossing time. Unlike traditional approaches that rely on wake features, our proposed method determines the crossing time by detecting the abrupt change in echo intensity along the time axis (i.e., the azimuth direction) of the time–frequency spectrum. Using this estimated timing, the target’s radial and azimuthal velocities are estimated. Subsequently, using the estimated velocity, the motion compensation of the moving target echoes is carried out through phase correction. Due to the difficulty in obtaining AIS data strictly synchronized with real SAR acquisitions, simulation data are initially utilized to verify the proposed method. The simulation results of moving ships with different velocities under three incidence angles demonstrate that the estimated errors of the radar radial and the azimuthal velocities generally remain below 0.1 m/s (2% relative error) and 0.5 m/s (5% relative error), respectively. Furthermore, after motion compensation, the azimuthal displacement caused by radial velocity is effectively corrected, restoring targets to their actual positions. Finally, the Level-0 raw data of ships acquired by Sentinel-1 SAR are applied to further verify the effectiveness of the method proposed in this paper.

## 1. Introduction

Synthetic aperture radar (SAR), as a microwave remote sensing technology, has been widely used for monitoring targets such as ships due to its capability to provide high-resolution imagery under all-day and all-weather conditions [1,2,3,4]. During the SAR imaging process, it is generally assumed that the target remains stationary throughout the synthetic aperture time. However, when the target is in motion, this fundamental assumption is violated. If a target has a radial velocity, it causes a shift in the Doppler center frequency. Since SAR imaging relies on the Doppler frequency to determine the azimuthal displacement of the target in the final image. Moreover, if the target has an azimuthal velocity, it disrupts the coherent phase accumulation during the aperture synthesis process, leading to improper focusing of the echo signals. As a result, moving targets typically exhibit azimuthal shifts and defocusing effects in the SAR imagery [5,6,7,8,9].

The current mainstream motion compensation methods are divided into two types, which are parametric methods and non-parametric methods [10,11,12]. The parametric motion compensation method estimates the motion parameters of the target to reconstruct the slant-range history relative to the radar, thereby achieving motion compensation. For the parameterized motion compensation method, more accurate estimation of motion parameters can make motion compensation more accurate. At present, significant research has been accumulated, encompassing a range of methodologies. Among them, wake-based velocity estimation methods have been proposed [13,14]. The wake broadly refers to the hydrodynamic effects generated by a moving ship on the water surface. In the context of this paper, it specifically refers to the Kelvin wake and the turbulent wake [15,16,17]. The motion of ships can be determined by analyzing the physical characteristics of the wake or by measuring the offset between the target’s imaged position and the wake’s position in the SAR image. Experimental results demonstrate that the wake-based method achieves a high degree of accuracy, confirming the effectiveness of this approach [18]. Furthermore, a specific relationship has been established between the wavelength of the Kelvin wake and the ship’s speed. It has been demonstrated that the speed can be estimated by analyzing the wavelength of the Kelvin wake. Experimental results have shown that the speed estimation error typically ranges from 4% to 9% for this method [19,20,21]. However, these methods are not applicable when the wake is not visible in the imaging results. The visibility of the wake depends on several parameters, including ship speed, radar polarization model, imaging resolution, and others [22,23]. In most cases, it is not possible to directly observe the wake associated with a ship target. For example, in the case of RADARSAT-2 data, the probability of detecting wakes associated with ships is no more than 5% [24]. As a result, some methods estimate the ship’s velocity by enhancing and extracting wake features [25].

In addition to wake-based methods, estimation methods based on the Doppler frequency of the echo have been developed. In 1971, R.K. Raney conducted a seminal analysis of the intrinsic relationship between target motion parameters and SAR imaging results [26]. During the azimuthal motion of the platform, target motion induces changes in its Doppler history, with radial velocity causing a shift in the Doppler center frequency. This will consequently lead to an azimuthal shift in of the target in the imaging results. Furthermore, the azimuthal velocity induces a change in the Doppler modulation frequency, which subsequently causes the target to appear defocused in SAR images. This theoretical framework has underpinned the development of numerous Doppler frequency-based velocity estimation methods [27]. Moreira and Keydel proposed a method for estimating the azimuthal and radial velocity components using the Doppler frequency rate and range migration [28]. In 1990, S. Barbarossa et al. proposed a method for estimating the motion state of a target in the time–frequency domain using the Wigner-Ville Distribution (WVD) method [29,30]. This study demonstrated the effectiveness of estimating target motion parameters using time–frequency analysis. Sparr noted that in some cases, there is no significant difference between the SAR images of moving and stationary targets, making it challenging to determine the target’s motion state based solely on the images. However, the time–frequency characteristics of the two exhibit significant differences. As research progresses, an increasing number of time–frequency analysis methods are being utilized for motion estimation [31,32]. In 2011, Jeong-Won Park et al. analyzed the errors in extracting target velocity parameters using the Wigner-Ville Distribution (WVD) method based on point target SAR simulations. They further validated the practical feasibility of the method through experimental studies using corner reflectors [33]. Among existing studies on extracting target motion parameters using time–frequency analysis, most simulated studies focus on point targets located at the scene center. Experimental studies are predominantly conducted using ground-based backgrounds and corner reflectors, which can be approximated as point targets. However, relatively few studies have addressed the estimation of velocity parameters for ship targets. Unlike the ground background, the ocean background is characterized by continuous dynamic motion. Ship targets are often large, spanning multiple range gates, and exhibit complex structures. All of these factors will have an impact on the analysis.

In this paper, we propose a velocity estimation and motion compensation framework for ship targets in maritime environments based on time–frequency analysis. A core feature of this method is the wake-independent estimation of the beam center crossing time. The raw SAR echoes are separated, and the estimated radial velocities are utilized to compensate for motion effects in the target echo components. It is important to distinguish this work from time–frequency analysis-based motion compensation in ISAR. While ISAR primarily addresses the rotational motion of non-cooperative targets for high-resolution imaging, our approach focuses on the translational motion, which is critical for wide-area maritime surveillance using standard SAR platforms. To ensure rigorous validation, the simulation model utilized in this study goes beyond conventional point-target simulations. The time-varying dynamic sea surface is constructed using the linear filtering method based on the ocean wave spectrum [34]. Subsequently, the backscattering coefficients are calculated using the Geophysical Model Function (GMF) to simulate the radar echoes [35]. Furthermore, a four-path model is employed to characterize multipath effects, encompassing both target-self multiple scattering and target-sea composite scattering [36]. This high-fidelity modeling ensures that the simulation data closely reflects the complexity of real-world vessel-sea scenarios, thereby compensating for the scarcity of AIS data synchronized with SAR acquisitions.

The following of paper is organized as follows: first, Section 2 theoretically analyzes the impact of target motion on Doppler parameters and SAR imaging results. It then briefly introduces the fundamental theory of the short-time Fourier transform (STFT) and the target velocity estimation method. Finally, a method for estimating the beam center crossing time of the target is proposed. Section 3 presents the results of the relevant analyses using both simulated and measured data. Section 4 provides the conclusions.

## 2. Moving Target Velocity Estimation and Motion Compensation

In this section, we present the velocity estimation method and its motion compensation scheme. Firstly, the influence of motion parameters on Doppler parameters of the echo is established. Then, the target velocity estimation method based on the time–frequency analysis and the translational motion compensation of the echo using the radial velocity estimates are presented.

### 2.1. Doppler Parameters of Moving Target’s Echo

**Figure 1:**
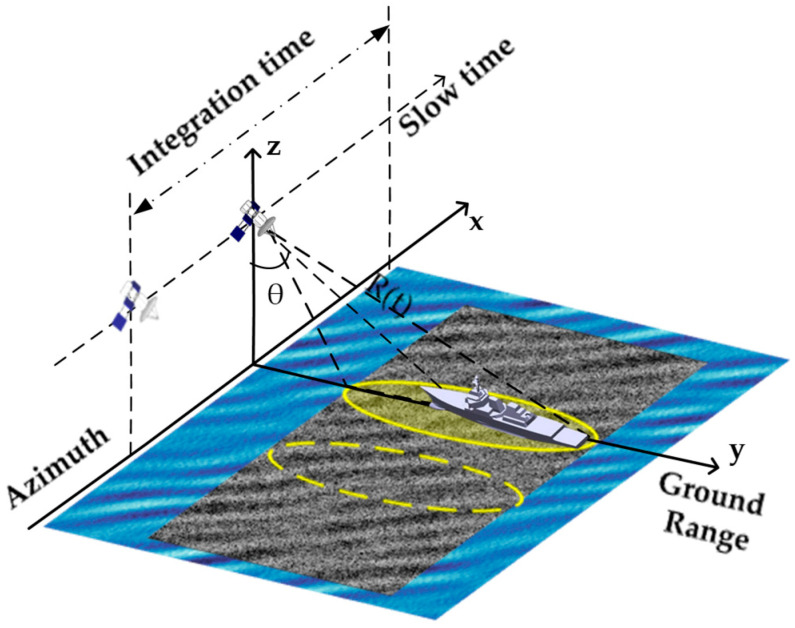
SAR geometry diagrams.

Figure 1 illustrates the SAR imaging geometry for surface and ship targets. Assuming stable platform motion, i.e., traveling at a constant velocity along the azimuthal direction on an ideal straight line trajectory, the instantaneous slant range R(t) for a moving target located at point $\left(x_{0},y_{0}\right)$ with velocity $\left(v_{x},v_{y}\right)$ and acceleration $\left(a_{x},a_{y}\right)$ can be simplified when the Earth’s curvature is neglected as [37]

(1) $$\begin{array}{cc} R(t) & ={\left[{\left(x_{0}+\left(v_{x}-V\right)t+\frac{a_{x}}{2}t^{2}\right)}^{2}+{\left(y_{0}+v_{y}t+\frac{a_{y}}{2}t^{2}\right)}^{2}+h^{2}\right]}^{1/2}\\ & \approx R_{0}+\frac{\left(x_{0}v_{x}+y_{0}v_{y}\right)-x_{0}V}{R_{0}}t+\frac{(v_{x}^{2}+v_{y}^{2}+x_{0}a_{x}+y_{0}a_{y}-2v_{x}V)+V^{2}}{2R_{0}}t^{2} \end{array}$$

Equation (1) performs a Taylor expansion of the instantaneous slant distance, ignoring the effect of higher order terms. V represents the radar platform velocity and t represents the slow time. $R_{0}$ represents the slant range at rest, which can be expressed as

(2) $$R_{0}=\sqrt{{x_{0}}^{2}+{y_{0}}^{2}+h^{2}}$$

where h represents the height of the platform. Based on Equation (2) the echo phase can be expressed as

(3) $$\phi \left(t\right)=\frac{4\pi R\left(t\right)}{\lambda }$$

where λ represents the electromagnetic wave wavelength. The Doppler center frequency can be estimated using the first-order term in Equation (1), as given by

(4) $$f_{dc}=f_{dc0}+\Delta f_{dc}=-\frac{1}{2\pi }\frac{d\phi \left(t\right)}{dt}=-\frac{2}{\lambda }\frac{x_{0}v_{x}+y_{0}v_{y}-x_{0}V}{R_{0}}$$

For conventional spaceborne SAR systems, the azimuth coordinate $x_{0}$ is typically several kilometers, whereas the range coordinate $y_{0}$ is determined by the incidence angle and orbital altitude. In our simulation, $y_{0}\approx 798$ km and $R_{0}\approx 1128.5$ km, assuming that the velocity components $v_{x}$ and $v_{y}$ are of comparable magnitude, the term $\frac{x_{0}v_{x}}{R_{0}}$ is found to be on the order of 10^−3^, while $\frac{y_{0}v_{y}}{R_{0}}$ is on the order of 10^−1^. Since the former is approximately two orders of magnitude smaller than the latter, given $\frac{x_{0}v_{x}}{R_{0}}\ll \frac{y_{0}v_{y}}{R_{0}}$, $f_{dc0}$ and $\Delta f_{dc}$ can be expressed as

(5) $$f_{dc0}=\frac{2}{\lambda }\frac{x_{0}V}{R_{0}}$$

(6) $$\Delta f_{dc}=-\frac{2}{\lambda }\frac{x_{0}v_{x}+y_{0}v_{y}}{R_{0}}\approx -\frac{2}{\lambda }\frac{y_{0}v_{y}}{R_{0}}$$

where $f_{dc0}$ is the Doppler center frequency at rest, and $\Delta f_{dc}$ is the Doppler center frequency shift induced by motion. Through the SAR imaging geometry relationship, we can obtain the relationship between the target velocity, and the coordinates in oblique and ground distance as $\frac{v_{r}}{v_{y}}=\frac{y_{0}}{R_{0}}$, substituting into Equation (6), where the relationship between the additional Doppler frequency $\Delta f_{dc}$ due to the target motion and the radial velocity $v_{r}$ can be obtained as

(7) $$\Delta f_{dc}=-\frac{2v_{r}}{\lambda }$$

The Doppler modulation frequency can be expressed by the second-order term in the Equation (1) as

(8) $$\begin{array}{cc} K_{am} & =-\frac{2}{\lambda }\left[\frac{(v_{x}^{2}+v_{y}^{2}+x_{0}a_{x}+y_{0}a_{y}-2v_{x}V)+V^{2}}{R_{0}}\right]=-\frac{2}{\lambda }\left[\frac{{\left(V-v_{x}\right)}^{2}+x_{0}a_{x}+v_{y}^{2}}{R_{0}}+a_{r}\right]\\ & \approx -\frac{2}{\lambda }\left[\frac{{\left(V-v_{x}\right)}^{2}}{R_{0}}+a_{r}\right] \end{array}$$

where $a_{r}$ is the radial acceleration, the Doppler modulation frequency is mainly affected by the radial acceleration $a_{r}$ and the azimuthal velocity $v_{x}$. When the target is stationary, i.e., $v_{x}=0,a_{r}=0$, the ideal Doppler modulation frequency can be obtained as

(9) $$K_{a}=-\frac{2}{\lambda }\frac{V^{2}}{R_{0}}$$

By analyzing the Doppler parameters of the moving target SAR signal, the target radial velocity causes a shift in the Doppler center frequency, which in turn creates an additional azimuthal offset in the imaging position. By affecting the Doppler modulation rate, the target azimuthal velocity causes incomplete compression of the azimuthal signal pulse, resulting in azimuthal resolution degradation, i.e., defocusing.

### 2.2. Time–Frequency Analysis and Velocity Estimation

The Fourier transform is an effective tool for analyzing smooth signals. However, as a global transformation, it fails to capture the time-varying characteristics of non-smooth signals. As a time–frequency analysis method, the short-time Fourier transform (STFT) captures time-varying signal characteristics by computing Fourier transform of windowed signal segments. Although the STFT cannot simultaneously achieve high-time resolution and high-frequency resolution due to the Heisenberg uncertainty principle, it does not suffer from cross-term interference, and it is more computationally efficient [38,39]. The time–frequency resolution provided by STFT is sufficient to meet the requirements of the analysis objectives in this paper, and the higher time–frequency resolution provided by methods such as WVD is relatively limited to help the subsequent calculations in this paper. Therefore, STFT is employed for time–frequency signal analysis in this study. For a deterministic signal $s\left(t\right)$ the STFT result can be expressed as [40]

(10) $$S_{STFT}\left(t,f\right)=\int_{-\infty }^{+\infty }s\left(\tau \right)h\left(\tau -t\right)e^{-j\omega \tau }d\tau$$

where $h\left(t\right)$ is the window function, the Kaiser window is employed in this research. Kaiser window can adjust the performance of the window function by adjusting the value of the parameter β, which gives Kaiser window the flexibility that other fixed window functions do not have, this also improves the overall flexibility of the algorithm. The length of the window determines the time resolution and frequency resolution of the STFT results, the longer the window, the higher the frequency resolution and the lower the time resolution; the shorter the window, the lower frequency resolution and the higher time resolution.

**Figure 2:**
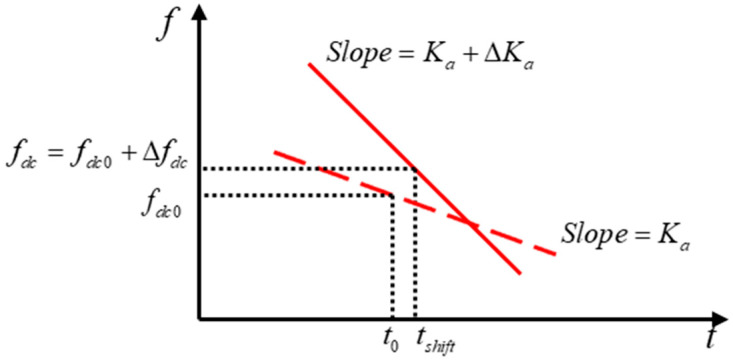
Schematic representation of the differences between stationary and moving targets in time–frequency domain. The red dashed line corresponds to the stationary target, while the solid line corresponds to the moving target.

As is evident from the preceding analysis of the Doppler parameters of the SAR signal, the radial velocity results in a shift in the Doppler center frequency, whilst the azimuthal velocity and radial acceleration lead to a change in the Doppler modulation rate. These effects are also reflected in the time–frequency spectrum of the signal. Figure 2 shows the schematic diagram of the time–frequency ridge offset caused by the target motion. As the target motion causes the time–frequency ridge to shift, the center frequency and slope after the shift correspond to $f_{dc}$ in Equation (4) and $K_{am}$ in Equation (8), respectively. Consequently, the time–frequency analysis of moving target echoes is performed by STFT to obtain the center frequency and slope of the time–frequency ridges. These can be used to invert the target velocity information based on the Doppler parameters of the moving target signals, as outlined in the previous section.

The STFT method is limited by an inherent trade-off between time resolution and frequency resolution, which results in the direct extraction of time–frequency ridges being afflicted by more pronounced jumps. In this study, the signal is first demodulated along the azimuthal direction to remove its frequency modulation. This process transforms the raw broadband signal into a narrowband signal. For clarity, the demodulation operator is defined as

(11) $$\phi^{-}\left(t\right)=\exp\left(-j2\pi \int_{0}^{t}\left(-\frac{2V^{2}}{\lambda R_{0}}u\right)du\right)$$

By defining the time domain signal before azimuthal compression after distance compression and distance migration correction as $s_{rcmc}\left(t\right)$, and then multiplying this signal with the demodulation operator, we can obtain the demodulated signal.

(12) $$s_{rcmc}^{d}=s_{rcmc}\left(t\right)\phi^{-}\left(t\right)$$

After conducting demodulation to remove Doppler modulation effects and conversion to narrowband signals, the time–frequency ridge discontinuity phenomenon is significantly mitigated.

The estimation of radial velocity from the time–frequency ridge’s center frequency relies on the fundamental relationship that the observed center frequency $f_{dc}$ comprises both the $f_{dc0}$ and the $\Delta f_{dc}$. It is necessary to remove the influence of the component $f_{dc0}$ to obtain the component $\Delta f_{dc}$ related to the radial velocity, and then the target radial velocity is calculated by Equation (7). Consequently, the problem of target radial velocity estimation is transformed into the estimation of $f_{dc0}$. From Equation (5), it can be seen that it is a function of $x_{0}$; where $x_{0}$ is the real position, which can be expressed as $x_{0}=Vt_{0}$, and $t_{0}$ is the beam center crossing time, which can be substituted into the Equation (5) to obtain the relationship as follows

(13) $$f_{dc0}=-K_{a}t_{0}$$

**Figure 3:**
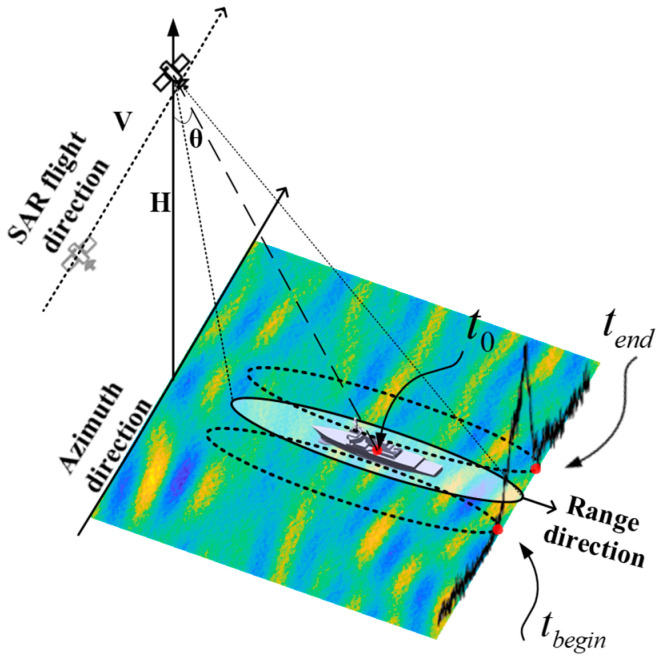
Schematic diagram of beam center crossing time calculation.

Therefore, a method is proposed in this paper to estimate the beam center crossing time and thus calculate the radial velocity; the detailed analysis process is as follows. In maritime radar observations, the backscattered intensity from ship targets significantly exceeds the sea surface clutter return. When the radar beam begins illuminating the target, the echo signal amplitude exhibits a significant increase due to enhanced backscattering from the target structure. Conversely, when the beam ceases illuminating the target, the echo signal amplitude decreases correspondingly. As shown in Figure 3, the initial moment of the target synthetic aperture is designated as $t_{begin}$, and the final moment is denoted by $t_{end}$. In the practical implementation, the signal boundaries $t_{begin}$ and $t_{end}$ are determined using an automated change point detection algorithm. The Root Mean Square (RMS) amplitude is selected as the statistical indicator. By minimizing the residual error of the segmented RMS levels, the algorithm precisely locates the abrupt changes in energy, defining the transitions between the noise floor and the target backscattering. Consequently, the beam center crossing time $t_{0}$ can be expressed as $t_{0}=\left(t_{begin}+t_{end}\right)/2$, based on both of the above, which can be obtained by substituting into Equation (13) as

(14) $$f_{dc0}=K_{a}\left(t_{begin}+t_{end}\right)/2$$

Based on Equation (14) and the corresponding time–frequency ridge, we can find the target radial velocity by using Equation (7).

The estimation of the target azimuthal velocity is also based on the time–frequency ridge. This deviates from the ideal modulation frequency in Equation (9) when there is an effect of the azimuthal velocity as well as the radial acceleration. This study focuses on surface vessel targets exhibiting accelerations below 0.2 m/s^2^, characteristic of typical maritime navigation scenarios. During conventional synthetic aperture time intervals, the velocity variation remains negligible. Therefore, this analysis disregards radial acceleration effects and assumes constant radial velocity throughout the aperture. Consequently, the Doppler chirp rate depends solely on the target’s along-track velocity, which is expressed as

(15) $$\Delta K_{a}=K_{a}-K_{am}=\frac{2}{\lambda R_{0}}\left(-2Vv_{y}+v_{y}^{2}\right)$$

The slope of the time–frequency ridge is obtained by linear fitting, and the deviation from the ideal Doppler modulation frequency is calculated in $\Delta K_{a}$. This is then substituted into Equation (15) and solved by the quadratic equation to find the target azimuthal velocity. However, the influence of azimuthal velocity on Doppler chirp rate is modulated by both platform velocity and slant range, whose magnitudes significantly exceed the target’s along-track velocity. As a result, the estimation accuracy of azimuthal velocity proves inferior to that of radial velocity.

### 2.3. Motion Compensation

The preceding Doppler analysis demonstrates that azimuthal displacement artifacts in SAR imagery arise from uncompensated Doppler shifts generated by target radial motion. Therefore, the phase compensation of the signal in the time domain can be performed based on the previous estimation result $\Delta f_{dc}$, and the compensation function is defined as

(16) $$h_{dc}=\exp\left(-j2\pi \Delta f_{dc}t\right)$$

Then the compensated signal $s_{mc}\left(t\right)$ can be expressed as

(17) $$s_{mc}\left(t\right)=s_{rcmc}\left(t\right)h_{dc}$$

Maritime SAR scenes exhibit dynamic sea surface movements while potentially containing static features such as islands or coastal structures. These background elements do not necessitate phase compensation. Even when disregarding background contributions, the required phase compensation terms vary significantly across targets with differing motion states in multi-target scenarios.

In summary, the processing of the bulk echo is incapable of meeting the demand for motion compensation in complex imaging scenarios. Consequently, signal decomposition based on motion state differentiation becomes essential, as it can independently compensate for each distinct motion component. The separation of signals with differing motion states in the time domain is challenging. Correspondingly, different motion states exhibit distinct spectral characteristics, with each dynamic regime occupying a unique frequency band in the Doppler domain. Frequency domain filtering as a separation method is a viable proposition. However, target motion with radial or azimuthal velocity components induces time-varying Doppler frequencies, rendering conventional frequency-domain analysis inadequate for characterizing these non-stationary signal components. For signals exhibiting such non-stationary behavior, the filter bandwidth must be sufficiently wide to encompass the complete time-varying spectral content of the signal component. Concurrently, an increased bandwidth inevitably incorporates additional signal components, but the time–frequency domain is better suited to reflect the time-varying characteristics of the signal. Therefore, time-varying filtering in this domain enables the separation of non-stationary signal components by adaptively tracking their instantaneous frequency characteristics. It is known that $S_{STFT}\left(t,f\right)$ denote the time–frequency representation of the echo signal $S\left(t\right)$. The filtered signal $S_{filt}\left(t,f\right)$ can be expressed as

(18) $$S_{filt}\left(t,f\right)=S_{STFT}\left(t,f\right)\cdot H\left(t,f\right)$$

Among them, the time-varying window function $H\left(t,f\right)$ is constructed to track the instantaneous Doppler centroid $f_{dc}$ of the target

(19) $$H\left(t,f\right)=\exp\left(-\frac{{\left(f-f_{dc}(t)\right)}^{2}}{2B_{f}^{2}}\right)$$

where $B_{f}$ represents the effective bandwidth of the filter, adjusted to preserve the target’s spectral energy while rejecting the surrounding clutter.

Following component separation, motion compensation is individually applied to each signal constituent using its specific motion parameters, after which the processed components are recombined for final imaging.

**Figure 4:**
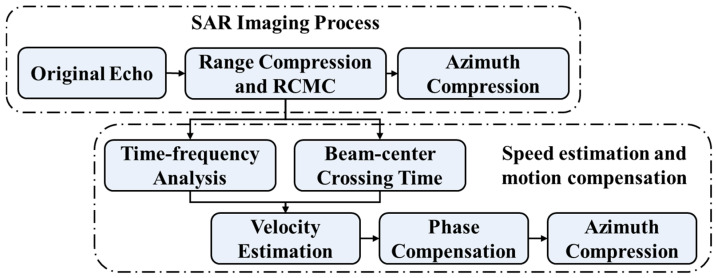
Flowchart of the algorithm.

Based on the above theories, Figure 4 shows the overall brief flowchart of the proposed algorithm. The upper branch of the flowchart illustrates the conventional SAR imaging workflow, while the lower branch depicts the proposed imaging framework which incorporates velocity estimation and motion compensation. In the subsequent analysis, we systematically compare the imaging results of these two approaches under various target velocities and sea states.

## 3. Experiments and Analyses

In this section, the feasibility of the aforementioned velocity estimation and motion compensation method is verified based on the surface ship SAR imaging simulation flow. Furthermore, the feasibility of applying the method to real scenarios is analyzed in preliminary terms. All experiments are implemented based on MATLAB 2022b software. Some of the starboard SAR simulation parameters are shown in Table 1.

### 3.1. Simulated Data Analysis of Radial Velocity Estimation

Using simulated SAR echo data of maritime vessels, we first apply range compression and range cell migration correction (RCMC), obtaining the time-domain signal. An alternative approach involves azimuthal decompression of Single-Look Complex (SLC) data, effectively reversing the SAR focusing process to reconstruct the raw signal phase history. This study systematically analyzes the distinct impacts of radial and azimuthal velocity components on SAR imaging quality when these motion vectors occur in isolation. In addition, the feasibility and accuracy of the velocity estimation method as described above are investigated.

**Figure 5:**
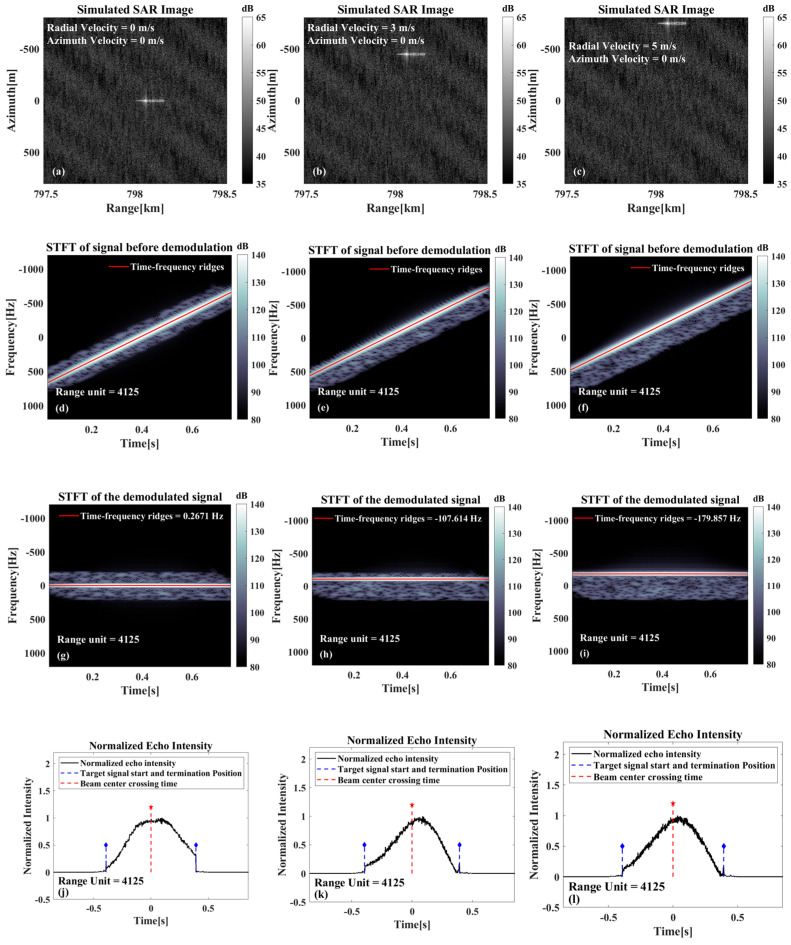
Relevant results only exist for the radial velocity case: (**a**–**c**) Imaging results at different radial velocities. (**d**–**f**) Time–frequency analysis results before azimuthal demodulation. (**g**–**i**) Time–frequency analysis results after azimuthal demodulation of the echoes. (**j**–**l**) Schematic diagrams of the estimated moments of traversal of the target beam center.

As illustrated in Figure 5, the results of the analysis are shown under different radial velocities. Furthermore, Figure 5a–c illustrates the imaging results under different radial velocities. These results indicate a gradual increase in the azimuthal offset with radial velocity. This finding agrees with the theoretical analyses in the previous section. From the SAR image alone, it is difficult to determine whether the target has an offset caused by radial velocity. This issue persists in real SAR images, especially for ship targets on the sea surface. Without visible wake features in the image, confirming the target’s positional shift due to radial velocity becomes challenging. Figure 5d–f display the time–frequency analysis of the raw signal, while Figure 5g–i show the analysis after azimuthal demodulation. As is evident in Figure 5d–f, there are discernible slopes in the time–frequency ridges corresponding to the targets and in the background information of the sea surface. Since this calculation series accounts exclusively for radial velocity effects, the ideal slope value corresponds to the Doppler modulation frequency induced by the platform’s azimuthal motion. As illustrated in Figure 5g–i, the demodulation process in the azimuth direction cancels the Doppler frequency modulation, causing the time–frequency ridges (including those from both targets and sea surface background) to be essentially horizontal. The instantaneous frequency of the time–frequency ridges corresponds directly to the Doppler center frequency $f_{dc}$. Although the three targets exhibit distinct azimuthal displacements due to differing radial velocities, their common initial positions cause identical beam center crossing time.

As illustrated in Figure 5j–l, these show the schematic diagram of the beam center crossing time calculation results. As all three targets are located at the scene center, their determined beam center crossing time are analogous, and they are all in proximity to the t=0, which is consistent with the theory. When the $f_{dc0}$ component calculated based on the beam center crossing time $t_{0}$ is similar to the Doppler center frequency, it can be deduced that there is no azimuthal shift phenomenon and almost no radial velocity. Conversely, if a deviation between the two is observed, it is indicative of an azimuthal shift phenomenon and the influence of radial velocity. The radial velocity of the target can then be calculated based on the deviation value.

**Figure 6:**
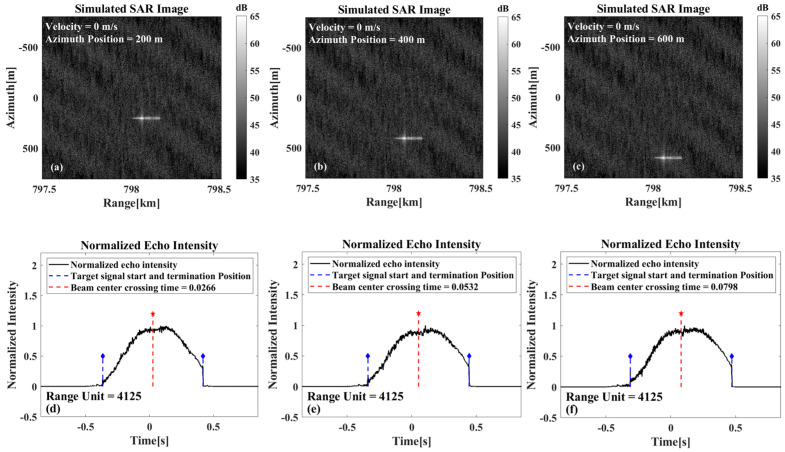
Stationary target beam center crossing time results for different azimuthal positions. (**a**,**d**) The imaging results and the azimuth normalized echo amplitude results at the azimuth position of 200 m. (**b**,**e**) The imaging results and the azimuth normalized echo amplitude results at the azimuth position of 400 m. (**c**,**f**) The imaging results and the azimuth normalized echo amplitude results at the azimuth position of 600 m.

As illustrated in Figure 6, these show the imaging results and beam center crossing time estimations for stationary targets at different azimuth positions. Based on the estimated crossing time, the derived azimuth positions are 200.83 m, 401.66 m, and 602.49 m, respectively. It is evident from the analysis that the calculated azimuthal position demonstrates accuracy with respect to the true position, and that the $f_{dc0}$ can be calculated based on this method.

**Figure 7:**
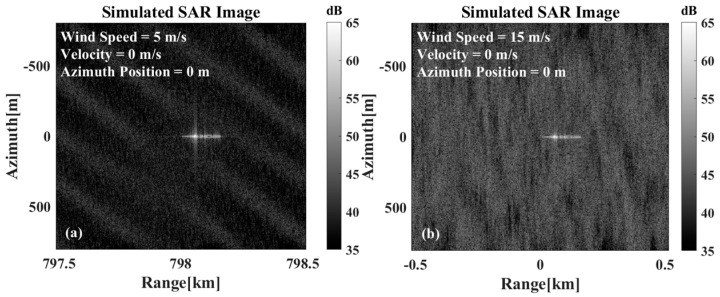
SAR imaging results under different wind speeds: (**a**) The wind speed is 5 m/s at 10 m above the sea surface. (**b**) The wind speed is 15 m/s at 10 m above the sea surface.

Since the calculation of the beam center crossing depends on the abrupt change in the target echo intensity compared with the sea clutter intensity, the influence of the sea clutter on the calculation of the beam center crossing time is briefly analyzed. Figure 7a,b show the imaging results when the wind speed is 5 m/s and 15 m/s ten meters above the sea surface, respectively. Figure 7 demonstrates that as the wind speed increases, the sea surface to target contrast decreases significantly in the SAR imaging results. This observation confirms that high sea state conditions adversely affect the beam center crossing time calculation, as theoretically analyzed in this study.

**Figure 8:**
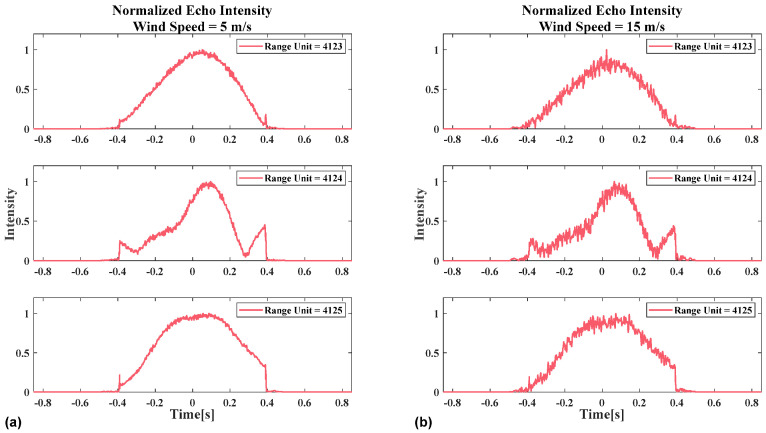
Schematic diagram of the azimuth normalized echo amplitude for different range gates and different wind speeds: (**a**) The wind speed is 5 m/s at 10 m above the sea surface. (**b**) The wind speed is 15 m/s at 10 m above the sea surface.

Figure 8 shows the schematic diagram of the normalized intensity of azimuthal echo at different range gates for the two wind speeds in Figure 7. First, analysis of the results from different range gates at identical wind speeds in Figure 8a reveals that the beam center crossing time calculation difficulty varies across range gates, even under low sea state conditions. Comparison of identical range gate echoes under different wind speeds in Figure 8a,b demonstrate that as wind speed increases, sea clutter intensity rises significantly. In some range gates (e.g., the 4123 range gate echo in Figure 8b), abrupt intensity variations in the echo signal could not be detected. In this scenario, calculating the beam center crossing time relying solely on a single range gate echo may lead to significant errors. In practice, to enhance robustness, echo signals from multiple range gates are averaged, and the estimation is subsequently performed on the resulting mean profile.

In real SAR systems, the incidence angle is not constant, whether it is the sea background, the target, or the composite scene; the backscattering characteristics will change significantly with changes in incidence angle, so it is necessary to analyze the performance stability of the algorithm under different incidence angle conditions. This study evaluates the robustness of this method with respect to the variation in the incidence angle.

**Figure 9:**
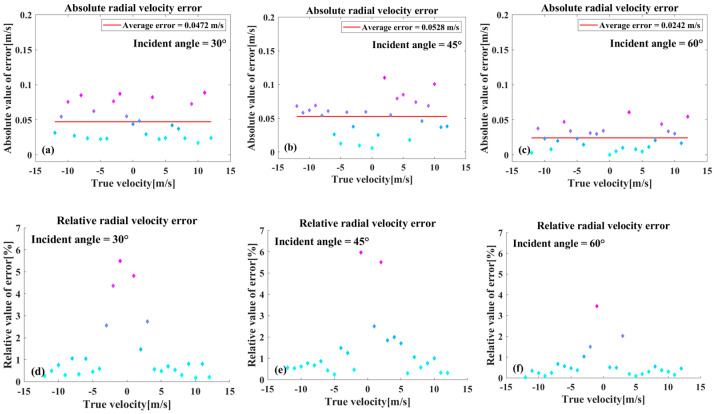
(**a**–**c**) A representation of the absolute values of the radial velocity estimation errors. (**d**–**f**) A representation of the relative values of the same errors.

Figure 9 presents an analysis of the discrepancy between the estimated radial velocity and the reference value at three incidence angles. As illustrated in Figure 9a–c, the absolute estimation error remain below 0.1 m/s at the three tested incidence angles. As radial velocity increases from −15 m/s to 15 m/s, the absolute error in radial velocity estimation oscillates about the mean value, showing no statistically significant correlation with the present radial velocities. As illustrated in Figure 9d–f, for radial velocities below 5 m/s, the maximum relative error can exceed 6%, and the fluctuation can be more pronounced. As radial velocity increases, the relevant error stabilizes, and the phenomenon is attributable to the intrinsic properties of relative error in velocity measurement. The mean values of the errors at incidence angles of 30° and 45° are similar, while the mean values of the errors at incidence angles of 60° are lower. However, the correlation remains statistically insignificant due to the small error. A comprehensive analysis of errors regarding both radial velocity and incidence angle reveals that there is no obvious correlation between the two. This analysis demonstrates that the method for estimating radial velocity is stable.

The method proposed in this paper is compared with the method of estimating the radial velocity of a ship based on the Kelvin wake wavelength mentioned in the introduction [16]. The maximum relative error of the proposed method is about 6%, and the maximum relative error of the method in the introduction is 9%. Therefore, although the accuracy of radial velocity estimation in this paper is shown to be an improvement, it is not significant. However, the method in this paper does not rely on the corresponding wake information of the ship for calculation, and has certain advantages in the scope of application.

### 3.2. Simulated Data Analysis of Azimuthal Velocity Estimation

**Figure 10:**
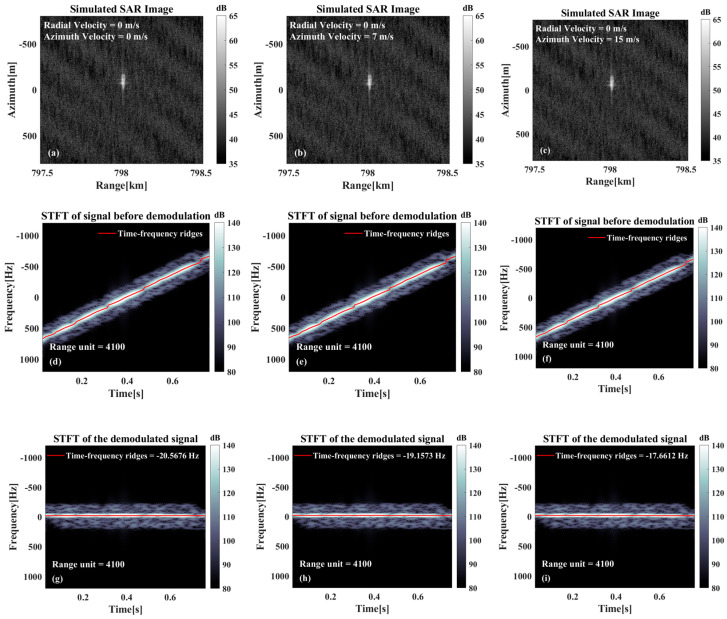

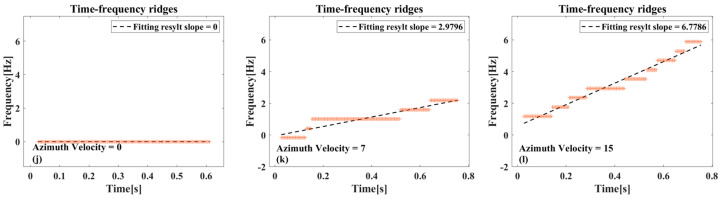
Relevant results only exist for the azimuthal velocity case: (**a**–**c**) Imaging results at different azimuthal velocities. (**d**–**f**) Time–frequency analysis results before azimuthal demodulation. (**g**–**i**) Time–frequency analysis results after azimuthal demodulation of the echo. (**j**–**l**) Time–frequency ridge extraction and fitting results.

Figure 10a–c show the imaging results under different azimuthal velocities. It is evident from the data presented in Figure 10 that the impact of azimuthal velocity on imaging resolution is negligible when compared to the effect of radial velocity. Even at the maximum azimuthal velocity (15 m/s), which is considered high, only a slight blurring is observed at the edge of the target. The rationale underpinning this phenomenon can be elucidated through a detailed examination of Equation (8), where the effect of azimuthal velocity on Doppler modulation frequency has been modulated by the platform velocity V and the slant distance $R_{0}$, which are numerically much larger than the Doppler modulation frequency. Under the simulation parameters of this study, taking a point target as an example, the 3 dB width of the imaging result is 14 m at an azimuth velocity of 15 m/s. This corresponds to a broadening of approximately 1.5 azimuth resolution cells on each side. Since ship targets are distributed targets occupying multiple resolution cells, such defocusing is not evident in the final image. Therefore, the reduction in azimuth resolution for ship targets caused by azimuthal velocity is not significant.

As illustrated in Figure 10d–f, the platform motion induces Doppler chirp rates significantly exceeding target-induced modulation effects by an order of magnitude. Utilizing the platform parameters outlined in this study, the azimuthal Doppler modulation frequency is estimated to be approximately −1819.6 Hz/s, and the modulation frequency change induced by a target with an azimuthal velocity of 10 m/s is 4.8168 Hz/s, which constitutes a mere 0.26% of the initial modulation frequency. Consequently, it is difficult to discern the modulation frequency change in the time–frequency ridges of the signals before demodulation. When the signal is subjected to azimuthally-based modulation at the ideal Doppler modulation frequency, the change in modulation frequency due to the target’s azimuthal velocity manifests as a residual slope, as illustrated in Figure 10g–i. As illustrated in Figure 10j–l, the time–frequency ridges of the demodulated signals and the corresponding fitting results reveal a salient feature. Specifically, when the azimuthal velocity is zero, the time–frequency ridge fitting slope is zero, indicating an absence of residual slope. However, as the azimuthal velocity increases, the slope of the fitted time–frequency ridge also increases, i.e., the residual slope increases.

**Figure 11:**
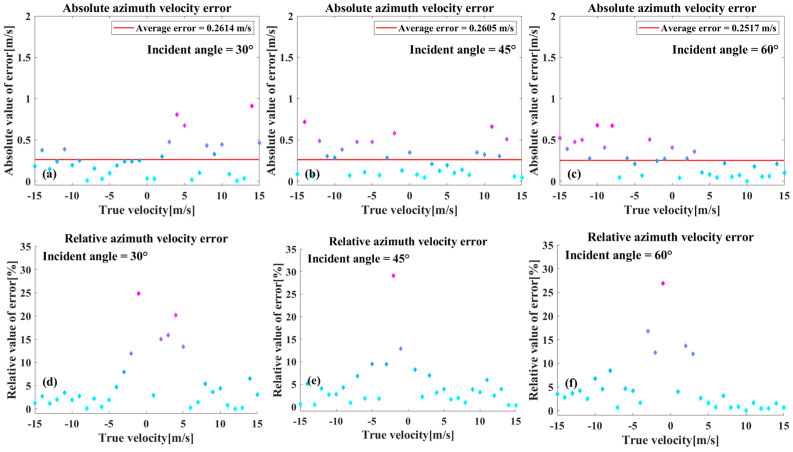
(**a**–**c**) A representation of the absolute values of the azimuthal velocity estimation errors. (**d**–**f**) A representation of the relative values of the same.

Figure 11 provides an analysis of the discrepancy between the azimuthal velocity estimates and the setup values for the three incidence angles. As illustrated in Figure 11a,b, the absolute errors demonstrate that the maximum value of the azimuthal velocity estimation errors at the three incidence angles is not more than 1 m/s, and the difference in the mean values is relatively small and does not show a more pronounced correlation with the incidence angle. As demonstrated in Figure 11d–f, the relative error exhibit a marked increase when the speed is small, reaching a maximum of 30%, before stabilizing as the speed increases. This behavior can be attributed to the same principle observed in the radial speed estimation.

The azimuthal velocity estimation error is significantly larger than the radial velocity estimation error. The radial velocity estimation error is typically maintained within 0.1 m/s; however, while the azimuthal velocity estimation error, can be controlled to within 0.5 m/s in most cases, it will exceed 0.5 m/s in a few cases, though it will not exceed 1 m/s. This phenomenon is influenced by several factors. Primarily, the interaction between the sea surface background, momentary motion, and frequency resolution can result in the time–frequency ridge spreading. This process hinders the precise extraction of the ridge frequency, leading to inaccuracies in the fitted ridge slopes and subsequent impacts on azimuthal velocity estimation outcomes. Secondly, in contrast to an ideal point target, a ship target presents extended geometric structures and multiple scattering centers. To enhance the fidelity of the simulation, the proposed SAR imaging model accounts for the multipath effect, which significantly influences radar return echoes from maritime targets. This effect arises from the coherent superposition of the ship’s composite scattering and the multipath contributions caused by ship-sea surface interactions, which jointly modulate the echo’s amplitude and phase. This also affects the accuracy and stability of the velocity estimation.

### 3.3. Simulated Data Analysis of Radial Velocity Motion Compensation

**Figure 12:**
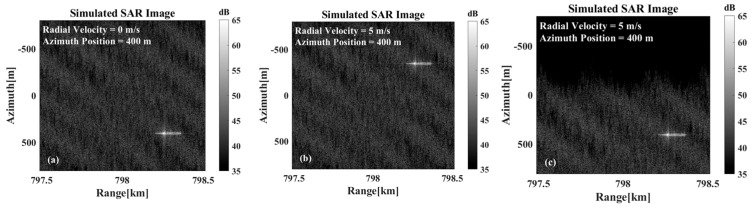
The imaging results were obtained under the following conditions: (**a**) The target was stationary. (**b**) The target was moving at a radial velocity of 5 m/s. (**c**) The overall echo was motion-compensated.

As evidenced by prior analyses, the image defocusing induced by azimuthal velocity remains inconspicuous. Consequently, this study focuses exclusively on motion compensation for the target’s radial velocity component. Figure 12a represents the static-target case, serving as the control group to demonstrate differences between uncompensated and motion-compensated imaging results. As illustrated in Figure 12b,c, the results before motion compensation and after motion compensation for the overall echo are demonstrated. The results demonstrate that the motion compensation processing corrects the imaged position of the target with radial velocity to its stationary position, thereby eliminating the azimuthal displacement caused by radial velocity. However, the global motion compensation applied to the composite echoes introduces shifts in the sea background. Since the sea surface exhibits inherent spatial statistical similarity, these background displacements alone have a negligible impact on the overall image quality.

**Figure 13:**
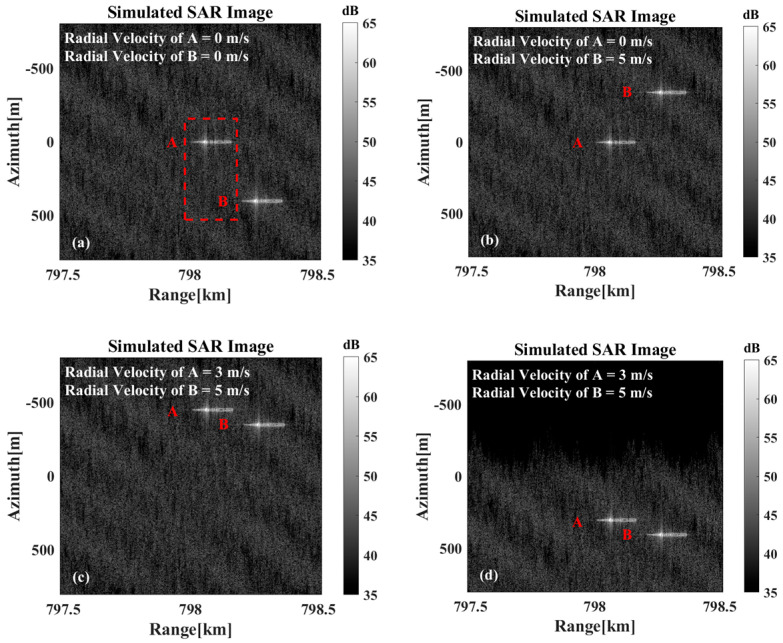
The imaging results are presented in the following instances: (**a**) When both targets are stationary. (**b**) Target A is stationary, while target B moves at a radial velocity of 5 m/s. (**c**) When target A moves at a radial velocity of 3 m/s and target B moves at a radial velocity of 5 m/s. (**d**) The motion-compensated results of the overall echoes with the velocity estimation results for target B.

However, when multiple targets are present within the imaging scene, the spatial statistical similarity between individual targets and the sea surface background differs from that of an isolated single target. Consequently, the background offset effect manifests with greater prominence in the imagery. As illustrated in Figure 13, the imaging simulation results demonstrate the concurrent existence of multiple targets. In Figure 13a, the two targets are depicted as at rest, while Figure 13b,c illustrate the results for the cases where one target is in motion while the other remains stationary, and where both targets are simultaneously in motion, respectively. As can be observed, compared to the scenario where both targets remain stationary, the relative positions of the two targets in the SAR imagery undergo significant changes, whether only one target is in motion or both are moving simultaneously. As illustrated in Figure 13d, motion compensation has been applied to the overall echo signal based on the velocity of Ship B. While Ship B has been correctly repositioned to its true location after compensation, both Ship A and the sea background exhibit noticeable shifts. Consequently, the SAR image still fails to accurately represent the true relative positional relationship between the two targets.

**Figure 14:**
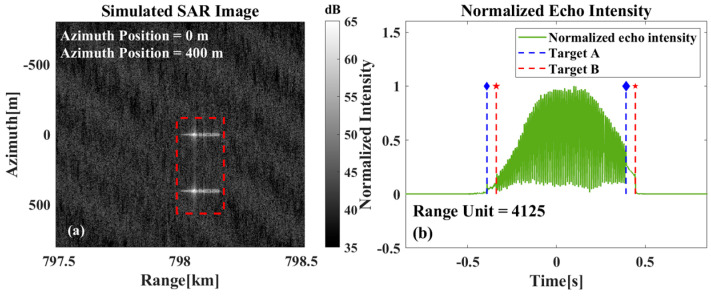
(**a**) Imaging results when two targets overlap in range direction, (**b**) Schematic representation of the estimated beam center crossing time of the multi-target.

For scenarios with multiple targets present simultaneously, they can be classified into two categories. The first category, as shown in Figure 13a, involves cases where the targets are incompletely overlapped in the range direction. In such cases, the beam center crossing time can be calculated directly based on the range gates where they do not overlap. Whereas, for the scenario shown in Figure 14a where the two targets are completely aligned in the range direction, special handling is required. Figure 14b shows the relevant result, from which it can be seen that when the two targets coincide in the range direction, the start time and termination time of the two targets can still be distinguished. Even if the start time and termination time of the two targets cannot be directly extracted, the start time of target A and the termination time of target B can still be extracted, and the beam center crossing time of the two targets can be calculated according to the synthetic aperture time of the system. Evidently, for the scene where three or more targets completely coincide, the echo information of the intermediate target is bound to be completely submerged.

**Figure 15:**
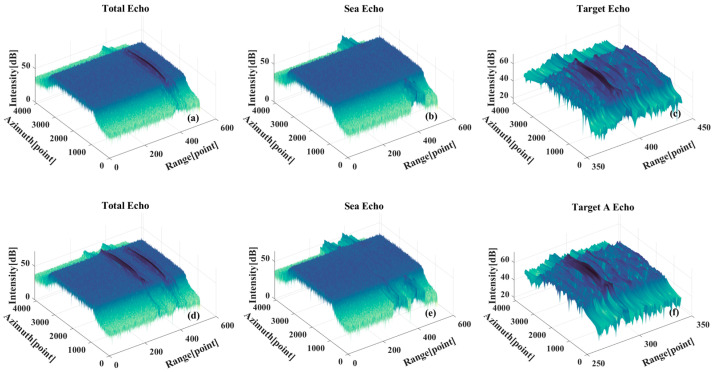
(**a**–**c**) Signal component separation results for the single-target case. (**a**) Global echo. (**b**) Sea surface echo component. (**c**) Target echo component. (**d**–**f**) Signal component separation results for the multi-target case. (**d**) Global echo. (**e**) Sea surface echo component. (**f**) Target echo component.

Figure 15 displays the outcomes of time-varying filtering processing, where the input signals have been pre-processed with range compression and range migration correction. It can be seen that the time-varying filter separates the target echo component and the sea surface echo component better, but the sea surface echo component still has some residual sidelobe effects. Figure 15d–f show the signal separation results for the multi-target case, where only the echo component of target A is shown here due to the similarity of the target echoes. Furthermore, the sea surface echo component depicted in Figure 15b is similar to the results of the single-target case.

**Figure 16:**
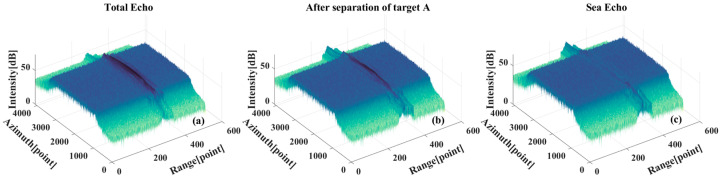
Multi-target signal separation results. (**a**) Total echo, including two targets and sea background. (**b**) The echo after separating one of the targets, including one target and sea background. (**c**) Sea surface echo after complete separation, containing only the sea background.

In the aforementioned multi-target scenario, the targets remain non-overlapping in the range direction. However, when multiple targets overlap in range, the situation becomes notably more complex. Under such conditions, multiple distinct ridges may emerge in the time–frequency domain. The filter is designed based on different time–frequency ridges and then separates the target echo components corresponding to the ridges. Figure 16 shows the separation results of two target signals overlapping in the distance direction. Figure 16a shows the overall echo, due to the complete overlap of the two targets in the range direction, so it is difficult to distinguish the existence of multiple targets in the results without orientation compression. Figure 16b,c show the echo after separating one of the targets and the surface echo after completely separating the targets, respectively. The residual target echo effect is clearly observable in the imaging results, manifesting as persistent artifacts at the target’s original position.

**Figure 17:**
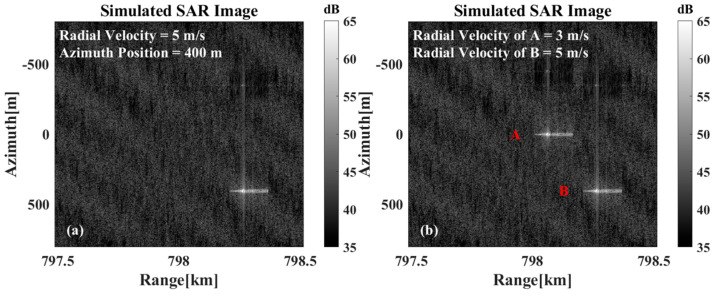
Motion compensation results after signal separation: (**a**) A single target moving at 5 m/s radial velocity, and the imaging results when the target is stationary are the same as in Figure 12a. (**b**) Target A moving at 3 m/s radial velocity, target B moving at 5 m/s radial velocity, and the imaging results when the target is stationary correspond to Figure 13a.

As illustrated in Figure 17, the outcomes of motion compensation for the signal components of the moving target are demonstrated. Figure 17a depicts the single-target case, and Figure 17b illustrates the multi-target case. As is evident from these, both cases can be more accurately adjusted to the true position of the target based on the estimated radial velocity-based motion compensation. In both scenarios, a shadow-blurring effect is clearly observable on the sea surface at the original target positions. This phenomenon arises because the filter window parameters in the time-varying filtering process critically determine target echo separation performance. During this process, a portion of the sea surface echoes are separated with the target echoes. After motion compensation, these sea clutter components exhibit azimuth displacement. This processing artifact generates shadow-like features at the original target locations. The temporal variation in the filter window parameters has been shown to cause information loss and degradation of sea surface echoes adjacent to the target’s initial imaging position. However, this approach demonstrates a negligible impact on both the sea surface imaging results in other regions and the target characterization. Moreover, it maintains superior preservation of original image features.

### 3.4. Validation Results Using Sentinel-1 Data

The previous section analyzes the velocity estimation and motion compensation algorithm using simulation data, and this section further verifies the algorithm of this paper based on the real data of the L0 level in Sentinel-1 SM mode. Table 2 shows the corresponding numbers of the data used. In the absence of prior knowledge regarding the ship’s motion parameters, we selected data containing visible wake features associated with the ship target. The tail track position is considered to be the real position of the target, and the accuracy of the radial velocity estimation and motion compensation algorithm is judged by whether the target position is restored to the tail track position after the motion compensation. It should be emphasized that the wake information serves solely as validation reference for motion compensation accuracy and does not participate in radial velocity estimation. The proposed method remains fully applicable to scenarios without ship-associated wake features.

**Figure 18:**
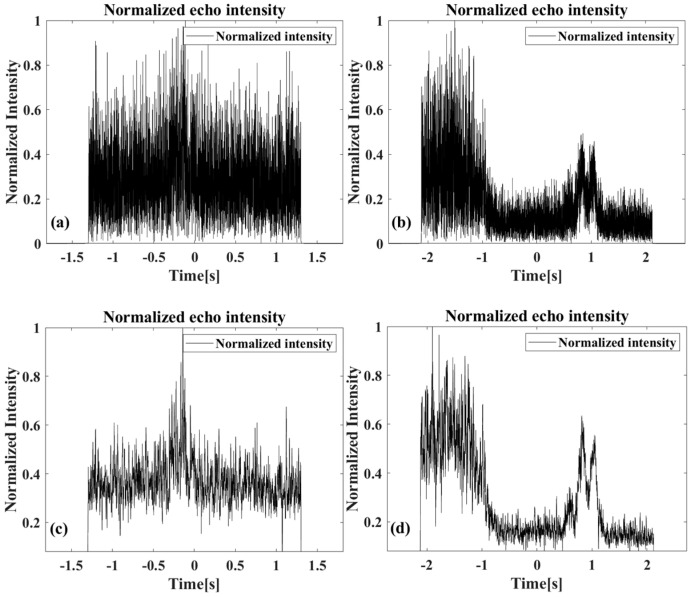
Normalized echo amplitude in the azimuth direction of Sentinel-1 data: (**a**,**b**) Schematic of azimuthal echo amplitude. (**c**,**d**) Results after sliding mean.

Figure 18 is the echo amplitude diagrams. For real data, the echo components are more complex. Taking the marine background as an example, which is the focus of this paper, strong sea clutter interference may occur. As shown in Figure 18a, the target echo strength is weak, and no significant increase in echo amplitude can be observed. In Figure 18b, although the elevated echo strength caused by the target is visible, the oscillation of the echo makes boundary delineation challenging. Figure 18c,d show the results after applying a sliding average. Compared to the unprocessed results, the echo trend becomes more apparent; however, the boundary values $t_{begin}$ and $t_{end}$ still cannot be accurately extracted.

**Figure 19:**
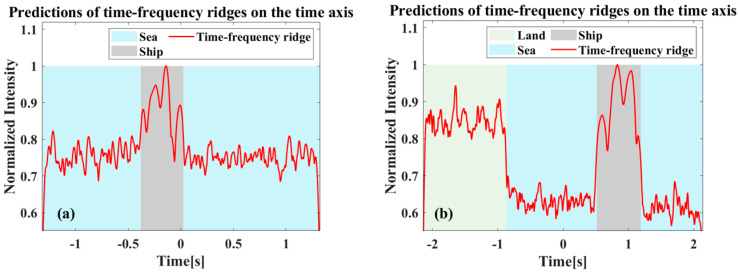
Time–frequency ridge projection to timeline results: (**a**) Corresponding to in Figure 18a,c. (**b**) Corresponding to in Figure 18b,d.

Therefore, real data can be combined with the start and end moments of the target’s time–frequency domain ridges to assist in the calculation. By projecting the time–frequency ridges onto the time axis and utilizing the amplitude of the abrupt changes, the corresponding points $t_{begin}$ and $t_{end}$ can be clearly delineated. The results corresponding to Figure 18 are given in Figure 19. For the target that was nearly submerged in the background echoes in Figure 18a, the processing enables clear observation of the time period during which the target appears, as shown in Figure 19a. This also demonstrates the applicability of the proposed algorithm in the background of strong sea clutter.

**Figure 20:**
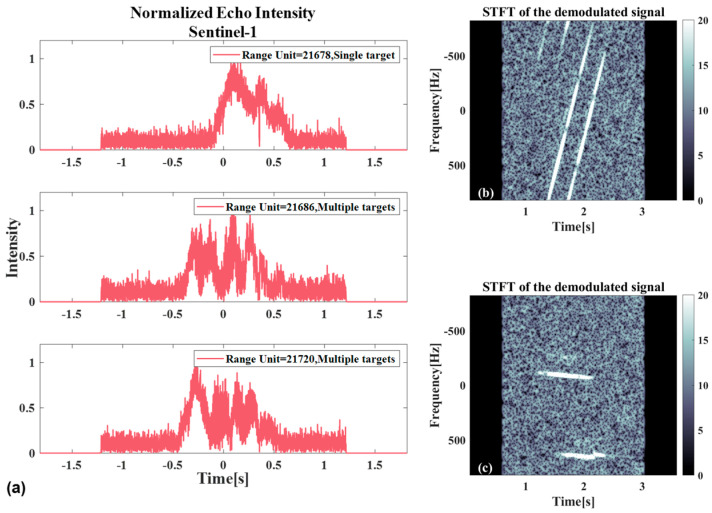
The relevant results when both targets exist simultaneously: (**a**) Schematic of the normalized azimuthal echo amplitude at different range gates, among them, the range gate 21,678 is not coincident, and the range gate 21,686 and the range gate 21,720 are coincident. (**b**) Time–frequency analysis results before azimuthal demodulation. (**c**) Time–frequency analysis results after azimuthal demodulation of the echo.

Figure 20 shows the relevant results in the presence of multiple targets. Figure 20a shows the azimuth normalized echo amplitude at different range gates, where only a single target exists in the results of range gate 21,678, and the other two range gate results are when two targets exist at the same time. When two targets exist at the same time, the calculation can be performed based on the corresponding results of the analysis results in Figure 14. Figure 20b shows the time–frequency analysis result of the echo from range gate 21,720, while Figure 20c presents the time–frequency analysis result after the echo has undergone demodulation processing. It can be observed that, regardless of whether demodulation processing has been carried out or not, the time–frequency ridges corresponding to the two targets do not overlap. Therefore, separation can be achieved through time-varying filtering.

**Figure 21:**
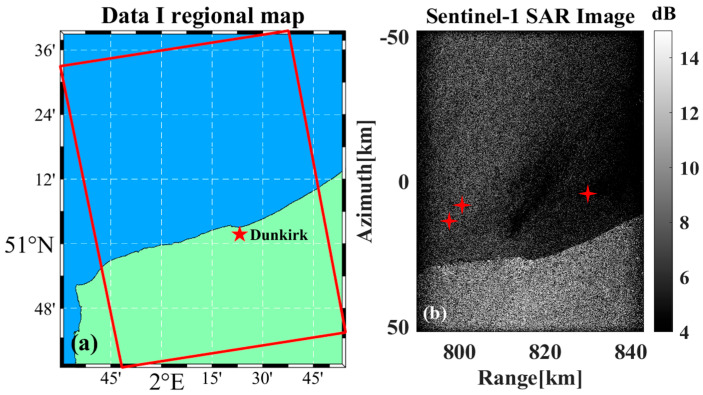
Data A-related results. (**a**) Map of the area. (**b**) Complete imaging results, the marked area indicates the selected ship targets for analysis, asterisks indicate the location of the ship targets.

Figure 21a,b show the schematic diagram of the selected Data A region and the complete data imaging results, respectively, as well as the three ship targets in the images that were selected as the motion compensation experimental data. It should be pointed out that the land part of the scene does not participate in the actual calculation. The sample targets selected in the analysis are all targets with a certain distance from the land and are not affected by the land echo. Here it is only to show the complete data imaging results where the selected ship target samples are located. The density of ship targets in the offshore channel or strait area is often higher; therefore the selected Sentinel-1 data all contain land.

**Figure 22:**
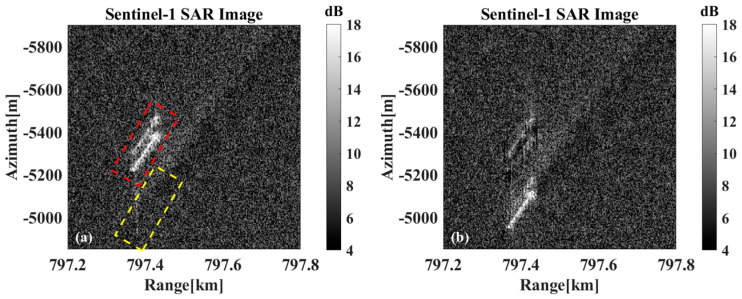

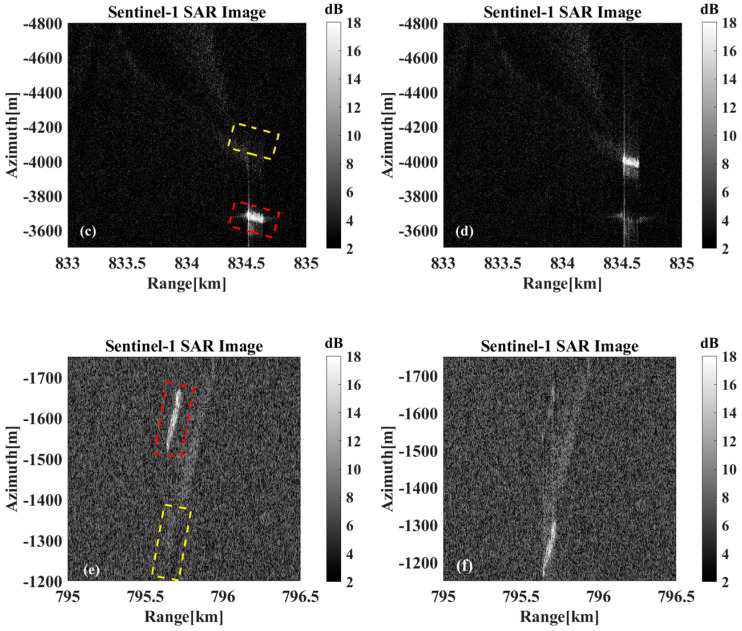
Results of motion compensation in Data A. (**a**,**c**,**e**) Before motion compensation, the red and yellow boxes indicate the imaging position before motion compensation and the actual position of the ships, respectively. (**b**,**d**,**f**) After motion compensation.

Figure 22a,c,e show the original imaging results without motion compensation, the red bounding boxes indicate the displaced target position due to azimuth shift, while the yellow bounding boxes mark the wake signature location corresponding to the target’s true geolocation, it can be observed that there is an obvious offset between the imaging position of the ship and the position of the tail track. The radial velocities are estimated to be 2.5871 m/s, 2.835 m/s, and 3.433 m/s, respectively, using the previous method. Motion compensation is performed on the target echoes with the estimated radial velocities, and Figure 22b,d,f are obtained. Afterward, the motion-compensated target returns to the position where the wake is located, proving the motion compensation’s effectiveness. At the same time, similar to the simulation results, the original position of the target after motion compensation also shows the phenomenon of residual shadow. The reason for this phenomenon is again due to the sidelobe effect of the strongly scattering target, where the sidelobe energy is not phase compensated and therefore remains at the initial position. In the actual application process, it is difficult to remove the influence of this part of the sidelobe energy.

**Figure 23:**
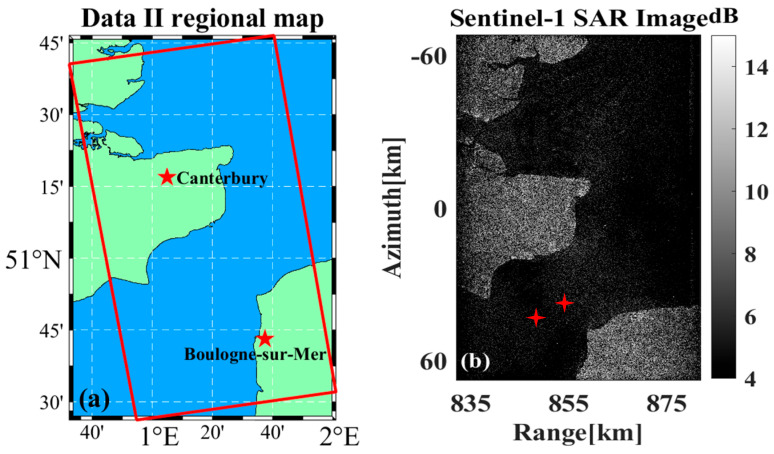
Data B-related results. (**a**) Map of the area. (**b**) Complete imaging results, the marked area indicates the selected ship targets for analysis, asterisks indicate the location of the ship targets.

Figure 23a,b show the schematic diagram of the selected Data B region and the complete data imaging results, respectively, with two ship targets in the image selected as the motion compensation experimental data.

**Figure 24:**
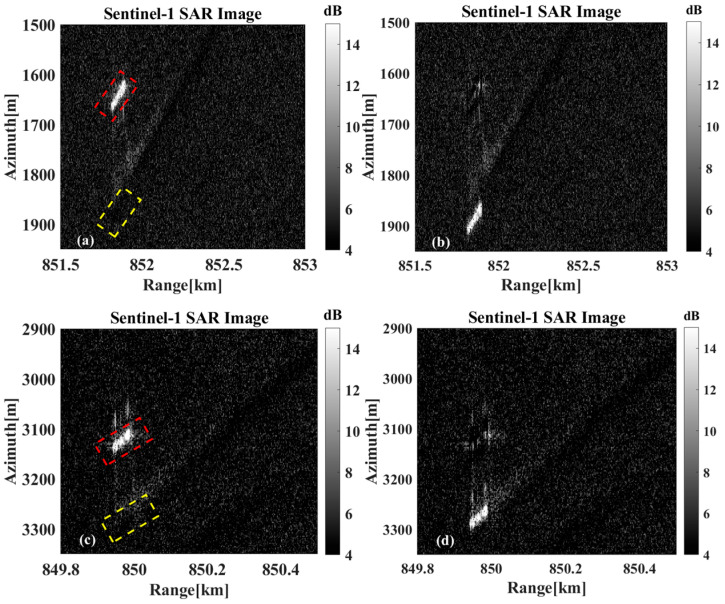
Results of motion compensation in Data B. (**a**,**c**) Before motion compensation, the red and yellow boxes indicate the imaging position before motion compensation and the actual position of the ships, respectively. (**b**,**d**) After motion compensation.

Figure 24a,c show the original imaging results without motion compensation, and Figure 24b,d show the original imaging results after motion compensation. The radial velocity estimates in the two sets of results were 3.8457 m/s and 2.3974 m/s. The target in Figure 24b is still offset from the location of the wake after motion compensation, which is caused by the radial velocity estimation error.

## 4. Conclusions

Moving targets typically exhibit azimuthal shifts and defocusing effects in SAR imagery, which severely degrade the detection performance of moving targets. To address this issue, this paper investigates the velocity estimation and motion compensation of ship targets in SAR imaging. First, the signal is converted to the time–frequency domain using STFT to extract the Doppler center frequency and Doppler modulation frequency from the target echo. Subsequently, the target velocity is calculated. A key step in this process is determining the beam center crossing time. To address this, we propose a method for estimating the crossing time of the radar beam center by detecting the abrupt change in echo intensity along the azimuthal time or the projection on time axis of the time–frequency spectrum. In motion compensation, the raw echo is first separated using time-varying filtering. Subsequently, motion compensation is applied to the separated target echo components based on the estimated radial velocity, thereby restoring the actual position in the SAR image.

In order to validate this method, the relevant experiments are conducted using simulation data and the Sentinel-1 SM mode Level-0 data. The accuracy of velocity estimation at three incidence angles is analyzed. The absolute error of the radial velocity estimation is generally less than 0.1 m/s. When the radial velocity is less than 3 m/s, the relative error can approach 6%, whereas for radial velocities greater than 3 m/s, the relative error remains stable within 2%. Compared to the radial velocity, the azimuthal velocity estimation errors are larger. In most cases, the absolute error of the azimuthal velocity can be kept within 0.5 m/s, though in a few instances, it may approach 1 m/s. For azimuthal velocities less than 3 m/s, the relative error can reach 30%, while for azimuthal velocities greater than 3 m/s, the relative error is mostly stabilized below 5%. The estimation errors for both radial and azimuthal velocities at the three incidence angles do not show significant variations. Therefore, the proposed velocity estimation method is not strongly correlated with the incidence angle. Based on the estimated radial velocity, the motion compensation of the echo of target is performed by phase multiplication. This effectively solves the azimuthal offset phenomenon induced by the target’s radial velocity. Sentinel-1 data are used to carry out relevant experiments to further verify the proposed method. In the analysis of the Sentinel-1 data, ship echo data with wake information is selected. Radial velocity estimation and motion compensation are performed using the proposed method. The results show that imaging positions of the target are consistent with positions of the corresponding wake, demonstrating the effectiveness of the proposed method. It should be noted that the wake information is not utilized in the radial velocity estimation or motion compensation processes; it is solely used as a reference for the true position of the ship to verify whether the azimuthal offset in the imaging results has been resolved. The method remains applicable even in cases where wake information is absent.

Although the proposed method effectively estimates target motion parameters and compensates for azimuthal displacement in moving target imaging, several limitations merit discussion. First, the method is based on a predefined target scenario and does not include target detection-related processes. Furthermore, both the time-varying filter performance and beam center crossing time estimation depend on the contrast between echo of the target and the background. Based on this, the proposed approach requires a certain SNR to ensure that such a contrast is evident. Therefore, future work will focus on establishing more explicit quantitative SNR thresholds and enhance the method’s applicability in low SNR scenarios. With the development of distributed SAR architecture [41,42], it has been proven to improve the accuracy of parameter estimation and imaging performance of moving targets, and the algorithm can be further extended to this kind of new SAR system in the future.

## Figures and Tables

**Table 1:** Sensor model parameters used for simulation.

Parameter	Value
Carrier frequency	5.4 GHz
Chirp bandwidth	100 MHz
Track height	798 km
Sampling rate	105.5 MHz
PRF	2407 Hz
Effective velocity	7550 m/s

**Table 2:** Sentinel-1 data number.

Data Number	Data ID
Data A	S1A_S3_RAW__0SDV_20150309T173239_20150309T173255_004958_006340_73FE
Data B	S1A_S4_RAW__0SDV_20150407T174049_20150407T174109_005381_006D5B_D361

## Data Availability

The data presented in this study are available on request from the corresponding author.
